# Therapeutics—Its Principles and Practice

**Published:** 1902-01

**Authors:** 


Therapeutics—Its Principles and Practice. By Horatio C. Wood, M. D., LL. D., Professor of Materia Medica and Therapeutics and Clinical Professor of Diseases of the Nervous System in the University of Pennsylvania, etc., Philadelphia. Eleventh edition, remodeled and in great part rewritten. By Horatio C. Wood and Horatio C. Wood, Jr., M. D., Demonstrator of Pharmacodynamics in the University of Pennslyvania. Pages, 850; price, $5.00. Philadelphia and London: J. B. Lippincott Company. 1900.

Wood's Therapeutics was the first work in this country in which a scientific classification was attempted. Improvement and additions have been made with each edition during the last twenty-five years and this the eleventh and last edition is the greatest volume of them all.

In the preface the author says that the principal changes in the work consist in making the various articles more closely conform to a uniform, carefully-thought-out plan of presentation; in putting in small type the descpritions of the general effects produced by drugs in the lower animals, minor discussions, and other matters of less importance than those considered in the general text; in cutting out certain discussions which were necessary in the earlier editions of the book, but have lost value at this time because of the general uniformity of professional opinion that has been reached in regard to their conclusions; in taking the references out of the body of the text and putting them in nonpariel type at the end of the various chapters; in the addition of articles on a number of new drugs, and in the insertion of the approximate metric equivalents of apothecaries' weights and measures used in giving the doses, etc.

